# Impact of Measurement Error on Testing Genetic Association with Quantitative Traits

**DOI:** 10.1371/journal.pone.0087044

**Published:** 2014-01-24

**Authors:** Jiemin Liao, Xiang Li, Tien-Yin Wong, Jie Jin Wang, Chiea Chuen Khor, E. Shyong Tai, Tin Aung, Yik-Ying Teo, Ching-Yu Cheng

**Affiliations:** 1 Department of Ophthalmology, National University of Singapore and National University Health System, Singapore, Singapore; 2 Singapore Eye Research Institute, Singapore National Eye Centre, Singapore, Singapore; 3 Department of Statistics and Applied Probability, National University of Singapore, Singapore, Singapore; 4 Saw Swee Hock School of Public Health, National University Health System, National University of Singapore, Singapore, Singapore; 5 Centre for Vision Research, University of Sydney, Sydney, Australia; 6 Division of Human Genetics, Genome Institute of Singapore, Singapore, Singapore; 7 Department of Medicine, National University of Singapore and National University Health System, Singapore, Singapore; 8 Duke-NUS Graduate Medical School, Singapore, Singapore; National Institute of Environmental Health Sciences, United States of America

## Abstract

Measurement error of a phenotypic trait reduces the power to detect genetic associations. We examined the impact of sample size, allele frequency and effect size in presence of measurement error for quantitative traits. The statistical power to detect genetic association with phenotype mean and variability was investigated analytically. The non-centrality parameter for a non-central *F* distribution was derived and verified using computer simulations. We obtained equivalent formulas for the cost of phenotype measurement error. Effects of differences in measurements were examined in a genome-wide association study (GWAS) of two grading scales for cataract and a replication study of genetic variants influencing blood pressure. The mean absolute difference between the analytic power and simulation power for comparison of phenotypic means and variances was less than 0.005, and the absolute difference did not exceed 0.02. To maintain the same power, a one standard deviation (SD) in measurement error of a standard normal distributed trait required a one-fold increase in sample size for comparison of means, and a three-fold increase in sample size for comparison of variances. GWAS results revealed almost no overlap in the significant SNPs (*p*<10^−5^) for the two cataract grading scales while replication results in genetic variants of blood pressure displayed no significant differences between averaged blood pressure measurements and single blood pressure measurements. We have developed a framework for researchers to quantify power in the presence of measurement error, which will be applicable to studies of phenotypes in which the measurement is highly variable.

## Introduction

In genome-wide association studies (GWAS), association between large number of single nucleotide polymorphisms (SNPs) and a trait measurement is computed and SNPs with strong associations will be replicated in a separate cohort. Non-differential measurement error in both genotyping and phenotyping reduces the power and hence increases the type II error to identify true associations in discovery cohorts. This decreases the efficiency of GWAS to produce findings in discovery that are less likely to be replicated in subsequent studies. Errors in genotype have been reduced through technological advances and stringent quality controls in SNP genotyping. Measurement and misclassification errors in case-control studies and measurement errors in exposure variables have been well studied[1]–[5]. However, to the best of our knowledge, there is only one paper evaluating the implications of measurement error in a continuous outcome in genetic analysis [6].

Performing power and sample size calculations allows researchers to manage cost of genotyping effectively. With recent discoveries made using web-based questionnaire for data collection [7], one may question the trade-off between sample size and accuracy of phenotype measurement to achieve a minimal level of statistical power. Using the asymptotic non-centrality parameter of the 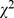 distribution, researchers have arrived at power and sample size formulas that account for misclassification error in case-control studies [8], [9]. Online programs PAWE-PH and PAWE-3D were also developed [10] and used to demonstrate that in case-control GWAS, there is substantial reduction in statistical power when diagnostic error increases, especially for lower allele frequencies and genotype relative risks [11]. Barendse [6] recommended checks at phenotype collection stage, but did not offer theoretical solutions in terms of power and sample size calculation.

In this study, firstly we quantified the power to identify genetic variants that affect the means and variability of quantitative traits in GWAS of unrelated individuals in the presence of measurement error, where measurement error was defined as the additional variation introduced to a “true” underlying phenotype. Secondly, we demonstrated the impact of measurement error on the pipeline of GWAS analysis in population-based studies. We presented real data analysis based on two phenotypes: age-related cataract and blood pressure to illustrate the impact of measurement error on GWAS discovery and on genetic replication studies.

## Materials and Methods

### Power to Detect Differences in Means

We used the following model to describe the phenotype:
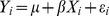
where 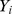 is the phenotype for the *i* ^th^ individual, 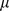 is the phenotype mean, 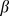 is the effect size of a SNP, 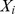 is the allelic dosage for the SNP, taking values 0, 1 or 2, and 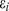 is the noise in the phenotype. We made the following assumptions:

1. The marker locus satisfies the Hardy-Weinberg equilibrium (HWE). Hence the genotype frequencies are computed based on *p*, the minor allele frequency (MAF). 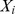 is dependent on *p* via a Binomial distribution.

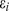
 follows a standard normal distribution, which can be achieved through standardization of a normally distributed phenotype.
3. SNP effects are additive. Without loss of generality, we let 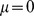. This can be easily extended when 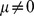 by centering the phenotype. Taking the previous assumption into account, the underlying true phenotype is standard normally distributed.

With measurement error 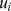, our model becomes:
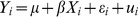
(1)where 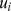 is normally distributed with mean 0 and variance 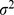, and independent of 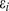.

The power for linear regression can be determined using the non-central F distribution, with non-centrality parameter (NCP) 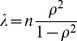 [12], where 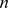 refers to the total sample size and 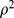 is the squared correlation coefficient. 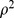 is computed as follows (Text S1):
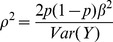
(2)


Without measurement errors, 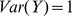. With measurement errors, 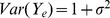. As 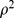 ranges from 0 to 1, we require 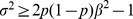. Since effect sizes in GWAS tend to be very small, this constraint is usually satisfied. Finally, power can be computed as 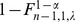, where 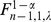 is the cumulative distribution function of the non-central F distribution with 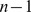 and 1 degree of freedom, non-centrality parameter 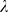, evaluated at the 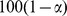 percentile of the F distribution.

### Power to Detect Differences in Variances

Following the framework described by Visscher and Posthuma [13], the underlying model of trait variance assuming there are no covariates is:
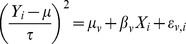
where 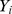 is the phenotype for the *i* ^th^ individual, 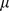 is the phenotype mean, 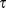 is the phenotype variance, 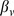 is the effect of a SNP, and 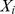 is as defined previously. 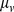 refers to the intercept of the regression of phenotype variability on genotype distribution and 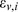 is the noise. We added a subscript of ν to denote that these variables are different from the model for comparison of means. In addition to the assumptions made for the previous model, we made the following assumptions:

1. The SNP has effect on phenotype variance but not the trait mean.
2. Phenotype is standard normally distributed in absence of heterogeneous variance.

We assume that 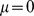, 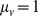 and 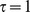 via standardization of a normally distributed phenotype. Hence, 
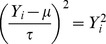
. The model with and without measurement error is summarized in Table 1. Using the same definition of the non-centrality parameter, we compute power with 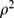 defined as (Text S1):
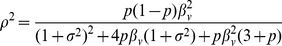
(3)


**Table 1:** Additive model for phenotype variances with and without measurement error.

Genotype	Frequency	Genotype Indicator	*E*(*y* ^2^)	*E*(*y* ^4^)	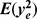	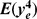
AA	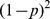	0	1	3	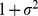	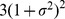
AB	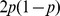	1	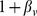	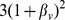	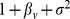	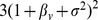
BB	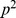	2	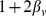	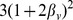	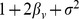	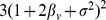

### Empirical Power Simulations

To verify our findings and assess the power of genetic association testing in the presence of measurement error, we carried out simulation studies under various scenarios. First, we simulated the genotypes 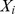 based on the Binomial distribution with probability *p*. For the comparison of phenotype means, the phenotypes were simulated using Equation 1, where the phenotypes have different means for different genotypes under the alternative hypothesis. For the test of difference in variances, the phenotypes were simulated under the normal distribution with mean 0 and variances based on Table 1, and the standardized and squared phenotype was used for testing. We performed 10 000 linear regressions for each simulation configuration and computed the empirical power, assuming 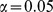. Configurations of model parameters were chosen to suitably represent the reality for future GWAS, where the effect sizes are expected to be very small and large sample sizes are required to detect the effects. Default parameters were *p* = 0.2, *n* = 15,000, 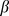 = 0.06 for the comparison of means and *p* = 0.2, *n* = 30,000, 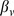 = 0.06 for the comparison of variances, and we varied only one parameter at one time. The R software version 2.14.2 was used for the simulations [14].

### Cost coefficients of Phenotype Measurement Error

We defined cost of phenotype measurement error as the percentage increase in sample size required to maintain a constant analytical power for an increase in measurement error. Following the framework of Edwards et al. [8], we set 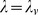, where 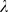 is the non-centrality parameter when there is no measurement error and 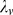 is the non-centrality parameter when there is measurement error. For comparison of phenotype means, we used Equation 2 with 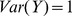 and 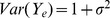 to obtain the following expression for the cost of phenotype measurement error:
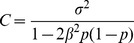



Similarly, for comparison of phenotype variances, we used Equation 3 and by letting 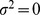 for 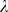, the following expression was obtained:
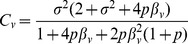



### Study Populations

The Singapore Malay Eye Study (SiMES) and Singapore Chinese Eye Study (SCES) are population-based cross-sectional epidemiological studies on eye diseases for residents of Singapore. Details of the study design and methodology have been reported and published elsewhere [15], [16]. In brief, a total of 4,168 Malay and 4,605 Chinese residents in the southwestern part of Singapore, aged 40 to 80 years old, were identified through age-stratified random sampling and were invited to participate in the study, for which 3,280 (response rate, 78.7%) Malays and 3,353 (response rate, 72.8%) Chinese underwent a detailed ocular examination. Ethics approval was obtained from the Singapore Eye Research Institute Institutional Review Board and all participants were provided with written informed consent in adherence to the Declaration of Helsinki.

### Phenotype Measurements

In the SiMES cohort, nuclear cataract was assessed using two methods: 1) the Lens Opacities Classification System III (LOCS III) [17] under slit lamp, and 2) the Wisconsin Cataract Grading System (Wisconsin System) based on lens photographs [18]. For LOCS III (decimal grade 0.1 to 6.9), participants went through slit lamp bio-microscopy where nuclear cataract was graded by multiple study ophthalmologists through comparison with standard photographs. For Wisconsin System (decimal grade 0.1 to 5.0), lens photographs were taken using a digital slit-lamp camera (model DC-1 with FD-21 flash attachment; Topcon, Tokyo, Japan) and grading was performed through comparison with standard photographs, at the University of Sydney by a single experienced grader, with adjudication by a senior ophthalmologist. A decimal grade was used if the severity of cataract was judged to be midway between two standards photographs. Higher accuracy and consistency is achieved with lens photographs graded by a single person. Hence, we assume that the Wisconsin System is the preferred grading system and deviation of the LOCS III grading from the Wisconsin System is regarded as measurement error.

In the Chinese cohort, blood pressure was measured according to a protocol used in the Multi-Ethnic Study of Atherosclerosis [19]. Blood pressure was measured twice, at an interval of 5 minutes. A third measurement was performed if blood pressure differed by more than 10 mmHg systolic or 5 mmHg diastolic. Blood pressure was taken as the mean between the two closest readings, which was assumed to be the “true” blood pressure value. The last measured blood pressure reading of an individual was assumed to contain measurement error for systolic and diastolic blood pressure (SBP_e_ and DBP_e_) and used for association testing in comparison with the “true” values (SBP and DBP).

### Genotyping and Data Quality Control

Genotyping of 3,072 and 1,952 samples in SiMES and SCES, respectively, was performed using Illumina Human610-Quad BeadChips (Illumina Inc.). A total of 620,901 SNPs were genotyped in each cohort. An additional 635 samples in SCES was genotyped using Illumina Human OmniExpress BeadChips with a total of 729,698 SNPs. Detailed quality control procedures for sample and SNPs were described elsewhere [20], [21]. In brief, samples were excluded based on the following conditions: (1) sample call-rates of less than 95%; (2) excessive heterozygosity; (3) cryptic relatedness; (4) gender discrepancies; and (5) discordant ethnic memberships. We excluded SNPs with (1) high missingness (>5%); (2) gross departure from HWE (*p* value <10^−6^) and (3) MAF <1%. Detailed quality control procedures for SCES samples genotyped on OmniExpress chips were provided in the supplementary materials (Text S2). After quality control, we have the following samples and SNPs available for analysis: 2,542 samples and 557,824 SNPs in SiMES, 1,889 samples and 538,408 SNPs in SCES on Illumina Human610-Quad BeadChips, and 615 samples and 633,783 SNPs in SCES on Illumina Human OmniExpress BeadChips.

### Real Data Analysis

For genome-wide analysis of nuclear cataract in SiMES, we used the nuclear cataract value from the worse eye, where a larger value indicates higher severity. Each phenotype was standardized by subtracting the mean and dividing over the SD of the phenotype. Association testing was performed on standardized nuclear cataract phenotype for comparison of means and squared-standardized nuclear cataract phenotype for comparison of variances. For genetic replication analysis, we analyzed 9 variants which showed significant associations with BP in East Asians [22]. We followed the analysis protocol used by Ehret et al. [22] for phenotypes DBP, DBP_e_, SBP and SBP_e_ in each cohort. In brief, linear regression analysis was performed assuming an additive model, adjusted for age, age-squared and body mass index (BMI), with medication corrected BP as the dependent variable. To account for batch effect of data from separate chips in SCES, meta-analysis was performed using an inverse-variance fixed effects model and a Bonferroni adjusted cut off of *p value = *0.0055 (0.05/9 tests) was used to control Type I error at 5%.

The PLINK software (version 2.0) [23] was used for association testing on nuclear cataract and blood pressure phenotypes. We assumed an additive genetic model where individual genotypes were coded according to the number of variant allele present. A trend test within a linear regression model was used to test the associations between phenotypes and SNPs.

## Results

### Power to Detect Differences in Means and Variances

**Figure 1:**
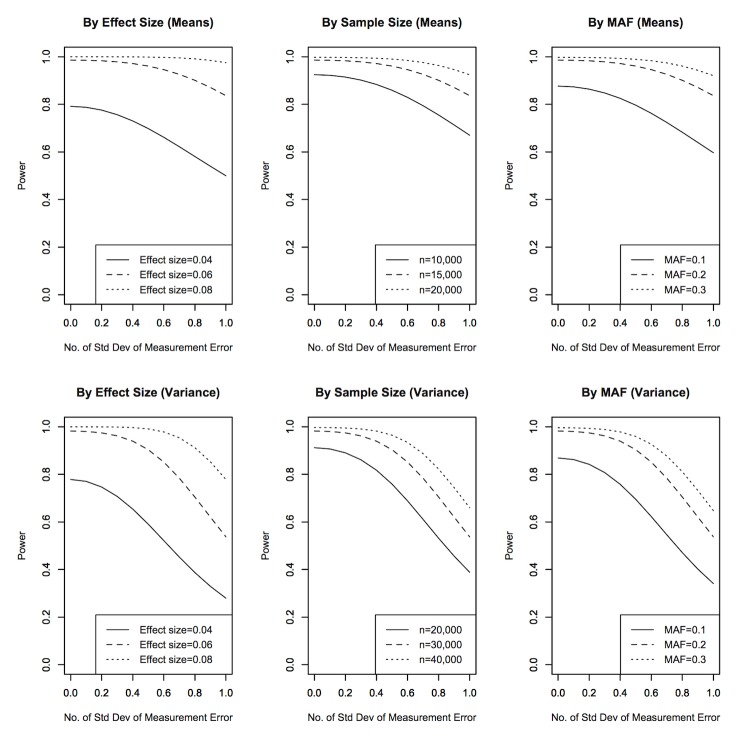
Impact of effect size, sample size and minor allele frequency on power. Measurement error is displayed in terms of the number of SD of the true phenotype (without errors). The top panel represents comparison of means and three configurations were considered with the rest of the parameters following the default configuration: *p* = 0.2, *n* = 15,000, 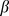 = 0.06. 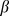 is interpreted as the change in the standardized phenotype for every increase in one effect allele. The bottom panel represents comparison of variances and three configurations were considered with the rest of the parameters following the default configuration: *p* = 0.2, *n* = 30,000, 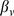 = 0.06. 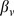 is interpreted as the change in the standardized and squared phenotype for every increase in one effect allele.

Figure 1 represents impact of effect size, sample size, and minor allele frequency on analytical power for comparison of phenotypic means and variances. For comparison of phenotypic means, there was substantial decrease in power when measurement error was larger than 0.6 SD of the true phenotype. Decreasing effect size to 0.04 (change in 0.02 SD per additional copy of the risk allele) had the most impact on power, dropping it by 20% even without measurement error. For comparison of phenotypic variances, the impact of measurement error on power was more significant. In most of the simulated configurations, there was substantial decrease in power when measurement error was larger than 0.4 SD. We also noted that an effect size of 0.06 with 0.7 SD of measurement error achieved equivalent power (78%) to an effect size of 0.04 with no measurement error.

To verify our findings, we compared the analytical power with the simulated power. The mean (SD) of absolute difference between the analytical power and simulation power for comparison of means and variances was 0.00169 (0.00195) and 0.00418 (0.00398) respectively. The maximum absolute difference for comparison of means and variances was 0.00941 and 0.0197 respectively.

### Cost Coefficients

For small effect sizes, *C* could be approximately equal to 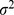. Hence the percentage increase in sample size ranged from 1% to 100% for measurement errors between 0.1 and 1.0 SD. For the analysis of heterogeneity of variances, the cost was almost three times higher as compared to the analysis of heterogeneity of means when the measurement error was equal to 1 SD of the phenotype (Table 2).

**Table 2:** Cost coefficients to account for measurement error.

Measurement Error 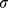 (SD)	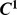	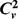
0.1	1.0	2.0
0.2	4.0	8.0
0.3	9.0	18.3
0.4	16.0	33.7
0.5	25.0	54.7
0.6	36.0	82.6
0.7	49.1	118.5
0.8	64.1	163.9
0.9	81.1	220.5
1.0	100.1	290.4

1 The following parameter values were used: *p* = 0.2, *n* = 15,000, 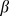 = 0.06.

2 The following parameter values were used: *p* = 0.2, *n* = 30,000, 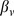 = 0.06.

### Replication and Genome-wide Association Testing Results

**Figure 2:**
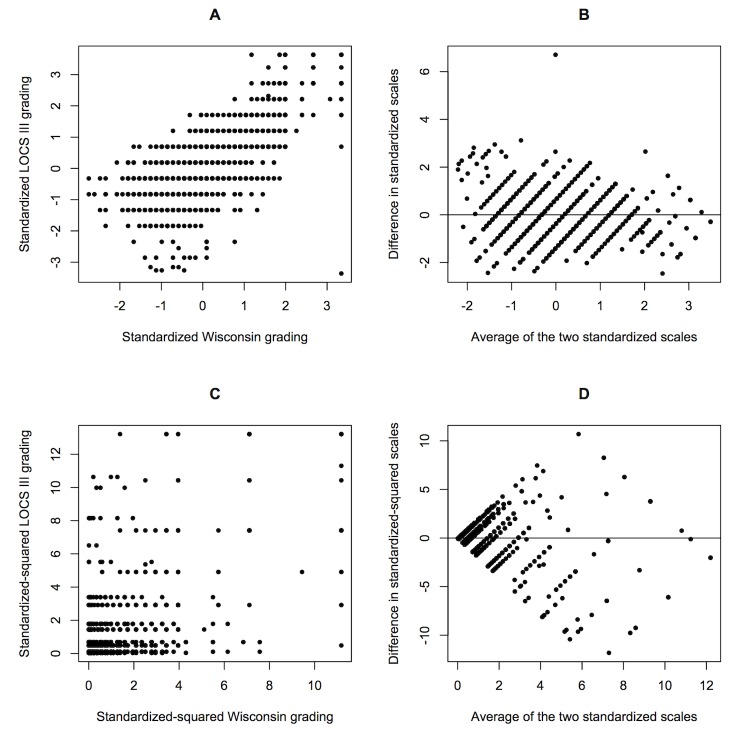
Deviation between Wisconsin System and LOCS III. (A) Standardized phenotype for comparison of means, (B) Bland-Altman plot of difference in standardized phenotype (Wisconsin System – LOCS III) against the average of the two, (C) Standardized and squared phenotype for comparison of variances, and (D) Bland-Altman plot of difference in standardized and squared phenotype (Wisconsin System – LOCS III) against the average of the two.

A total of 2,349 samples from SiMES with both genotype and phenotype data of Wisconsin System and LOC III grading were included for genome-wide testing. The measurements of nuclear cataract in SiMES varied substantially for some individuals (Figure 2), especially for the standardized and squared phenotype, which has SD of 1.52 and 1.80 for the Wisconsin System and LOCS III, respectively. The Pearson correlation between standardized phenotypes for the two grading systems was 0.71 while the correlation between the standardized and squared phenotypes was 0.56. The average measurement error was 0.0112, which corresponded to about 0.1 SD of the standardized Wisconsin System phenotype. Table 3 displayed the top SNPs (*p*<10^−5^) from both grading scales in the GWAS of nuclear cataract in a comparison of phenotypic means. None of the SNPs overlapped.

**Table 3:** Significant (*p* value<10^−5^) SNP in the GWAS of nuclear cataract (comparison of means).

SNP	Chr	Position(bp)	Effectallele	MAF	Effectsize	P value
Wisconsin System						
rs11184985	1	107,115,133	C	0.37	0.13	7.82×10^−6^
rs12133448	1	107,100,064	A	0.40	−0.13	5.94×10^−6^
rs1401830	1	107,068,638	A	0.37	0.13	9.09×10^−6^
rs777965	3	105,954,655	A	0.24	0.17	3.26×10^−7^
rs9985272	3	176,362,024	A	0.10	−0.22	3.73×10^−6^
rs6879319	5	117,214,194	G	0.37	−0.14	4.18×10^−6^
rs17066166	6	137,585,624	T	0.17	0.18	5.05×10^−6^
rs12931881	16	83,436,787	A	0.15	0.20	1.04×10^−6^
LOCS III						
rs4676323	2	107,164,560	G	0.13	0.19	7.81×10^−6^
rs1981845	5	53,734,292	A	0.29	−0.14	8.52×10^−6^
rs17072293	6	143,564,955	G	0.04	−0.40	4.43×10^−7^
rs6977512	7	39,471,584	T	0.26	0.15	5.52×10^−6^
rs917454	7	32,196,702	G	0.38	0.14	1.94×10^−6^
rs2160766	8	129,207,845	T	0.09	0.24	2.13×10^−6^
rs10760430	9	128,205,909	A	0.32	−0.15	4.48×10^−6^
rs11255087	10	7,441,387	G	0.03	−0.44	2.70×10^−6^
rs2724188	12	98,372,331	A	0.24	0.15	5.94×10^−6^
rs309427	15	82,932,421	G	0.03	−0.43	3.71×10^−6^
rs13038799	20	61,200,607	C	0.03	−0.44	1.86×10^−6^
rs3021272	22	38,730,950	G	0.03	−0.45	5.58×10^−8^
rs4145526	22	14,577,021	C	0.03	−0.42	3.25×10^−6^

MAF, minor allele frequency.

**Figure 3:**
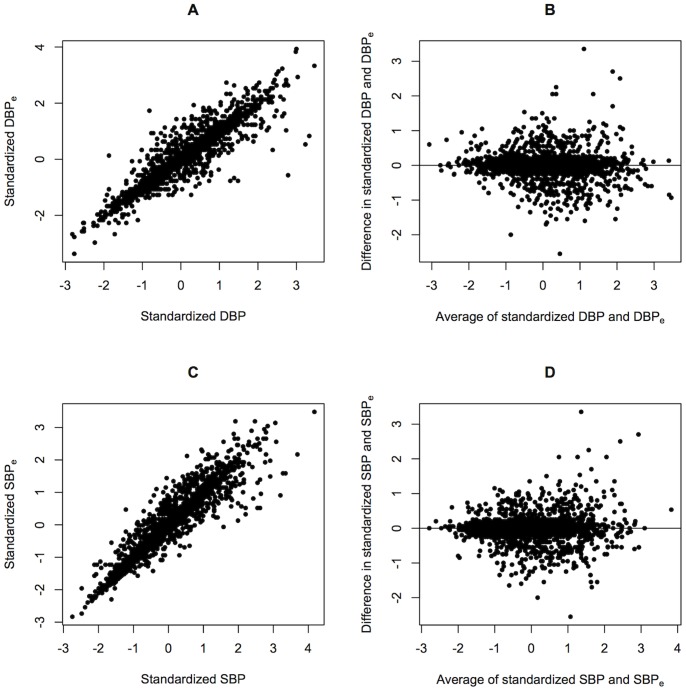
Deviation between blood pressure measurements. (A) Standardized phenotype for DBP, (B) Bland-Altman plot of difference in standardized phenotype (DBP – DBP_e_) against the average of the two, (C) Standardized phenotype for SBP, and (D) Bland-Altman plot of difference in standardized phenotype (SBP – SBP_e_) against the average of the two.

For genetic replication analysis, a total of 2,490 SCES samples with BP phenotype, age, gender, BMI information and genotype data were included. The Pearson correlations between DBP and DBP_e_ was high (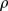 = 0.92) and the correlations between SBP and SBP_e_ was also high (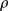 = 0.93). The average measurement error, defined as the mean absolute difference between the standardized values of the two measurements for systolic and diastolic blood pressure, was 0.251 and 0.252 respectively, which corresponded to about 0.25 SD of SBP and DBP (Figure 3). Table 4 showed the association results for the 9 variants previously found to influence blood pressure in East Asians. Variants replicated in DBP or SBP were also replicated in their error counterparts (rs633185 and rs17249754).

**Table 4:** Summary association results for 9 blood pressure SNPs.

						DBP		DBP_e_		SBP		SBP_e_	
Index SNP	Chr	Position	Gene	EA	MAF	Beta	P value	Beta	P value	Beta	P value	Beta	P value
rs1458038	4	81,383,747	FGF5	T	0.43	0.037	0.163	0.007	0.804	0.041	0.097	0.025	0.319
rs1173771	5	32,850,785	NPR3-C5orf23	G	0.32	0.031	0.283	0.046	0.127	0.019	0.469	0.027	0.326
rs11191548	10	104,836,168	CYP17A1-NT5C2	T	0.25	−0.0009	0.975	0.011	0.731	0.018	0.518	0.018	0.541
rs381815	11	16,858,844	PLEKHA7	T	0.14	0.050	0.186	0.037	0.342	0.097	5.8×10^−3^	0.086	0.016
rs633185	11	100,098,748	FLJ32810-TMEM133	C	0.48	0.101	1.6×10^−4^ *	0.111	5.3×10^−5^ *	0.087	3.9×10^−4^ *	0.089	3.9×10^−4^ *
rs17249754	12	88,584,717	ATP2B1	G	0.32	0.043	0.142	0.015	0.624	0.084	2.1×10^−3^ *	0.090	1.2×10^−3^ *
rs1378942	15	72,864,420	CYP1A1-ULK3	A	0.18	0.017	0.635	0.022	0.536	0.033	0.311	0.030	0.370
rs2521501	15	89,238,392	FURIN-FES	T	0.09	0.081	0.118	0.057	0.288	0.094	0.051	0.126	9.9×10^−3^
rs1327235	20	10,917,030	JAG1	G	0.45	0.042	0.120	0.048	0.084	0.017	0.503	0.009	0.719

EA, effect alleles.

* *p* value <5.5×10^−**3**^. Significance level was set at 0.05/9 = 0.0055.

## Discussion

We derived power calculations that take measurement error into account, which could be used for study design purposes. Using simulations, we verified our calculations and concluded that researchers may perform adequate power and sample size calculations for GWAS in the presence of phenotype measurement error. Recently, Yang, et al. discovered variants related to phenotypic variability of BMI in a GWAS setting [24]. Analyzing phenotypic variability could uncover presence of statistical interactions associated with the genetic variant that has not been account for. Various methods have been proposed for such analysis [13], [25]. Since measurement error affects the variability of phenotype, it is imperative that its impact on power should be studied closely. Hence, we developed the power analysis framework for comparison of both means and variances.

**Figure 4:**
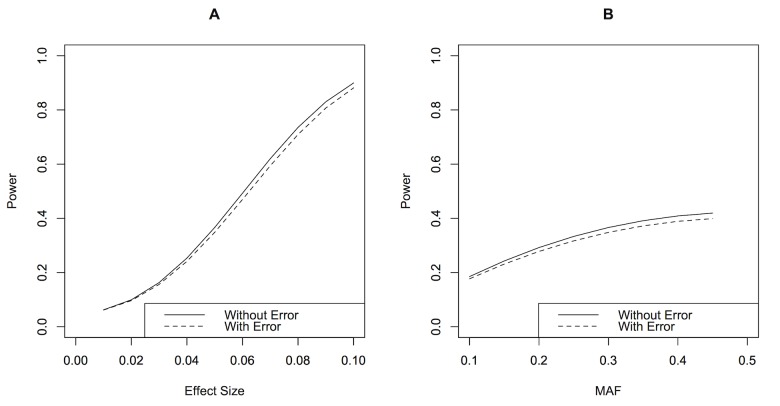
Comparison between power of GWAS of blood pressure measurements. (A) By effect size, the parameter values used were *p* = 0.3, *n* = 2,490. (B) By MAF, the parameters values used were 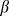 = 0.05, *n* = 2,490.

We used real datasets to demonstrate the impact of using different measurements of the same trait for GWAS. In the GWAS of nuclear cataract, our results displayed almost no overlap between the top SNPs associated with the two measurements. This finding was consistent with the results from Barendse [6] who also compared GWAS from two independent quantitative trait measurements of subcutaneous fat thickness in animals. In our replication study of BP, SNPs which replicated in the averaged BP measurements were also replicated in the single measurements. The minor differences suggest that failure to replicate is largely attributed to differences in genetic nature of the trait or false discoveries [26]. Based on our sample size, MAF and effect size range in our study, the power of GWAS of BP with a measurement error of 0.25 SD was almost identical to the power of GWAS of BP without measurement error (Figure 4). In the process of reaching these conclusions, we had assumed that the difference between trait measurements were only due to random errors. The Bland-Altman plots of the measurements in Figures 2 and 3 implies that the differences were more likely to occur at random and not due to systematic differences.

The impact on statistical power is much smaller in the presence of measurement error (of quantitative traits), compared to the presence of misclassification errors (of case-control status) for GWAS. We note that only as the measurement error exceeded 0.4 and 0.6 SD of the phenotype for comparison of means and variances respectively, the decrease in power became substantial. In current times, measurements prone to large errors have mostly been improved through technological advancements, or taking of multiple measurements and averaging them. While measurement error is not easily quantifiable in practice, we provide a framework to estimate measurement error using repeated measurements (Text S3).

In the National Cooperative Gallstone Study, it was reported that 7% and 17% of the variation in observed triglycerides and cholesterol values were attributable to errors respectively [27]. Depending on the settings or instruments used during phenotyping, measurement error in other studies ranged from 0.0035 to 0.63 SD of phenotype[28]–[31]. Knowledge of the impact of measurement error on statistical power can improve the efficiency of the data collection process with the optimal approach.

Our measurement error model has the same power as a classical measurement error model, where the error is in the independent variable instead of the dependent variable. The impact of measurement error under the classical measurement error model has been well studied in the area of econometrics and statistics [32]–[35] and results based on the linear and multivariate linear regression models could be extended to the GWAS framework. As estimates based on measurement error in the dependent variable are more innocuous than that based on the classical measurement error model, one need not apply bias-correction methods such as regression calibrations [36].

To reduce measurement error, simple methods such as trimming and winsorizing have been used to screen outliers [37]. Application of data trimming in GWAS context was performed by Barendse [6], where bivariate trimming resulted in improved correlation of two independent measurements of the same phenotype. Bollinger and Chandra, however, highlighted that only in the case where measurement error results in an upward bias in the regression coefficient could the simple outlier screening methods perform well without introducing more bias [38]. Another method in which measurement error can be reduced is through threshold-based sampling [39]. Using a Gaussian mixture model, the distribution of phenotype measurement can be described using three mixture Gaussian components, one for each genotype (AA, AB or BB). Samples with phenotype measurement that fall between two genotype distributions would likely be due to measurement error and subsequently be excluded from analysis. Although this method results in a reduction of sample size, there is a potential gain in power through decreased variability of phenotype. Power calculations for threshold traits with two categories (case-control) in association-based studies have been described by Gorden et al. and Purcell et al. [10], [40]. We suggest that if the power quantified based on our framework is low, apart from collection of additional samples, the sampling method based on mixture models could be a good choice for consideration.

In this work, we chose to compute power based on the simple linear regression framework and additive allele effects. We recognize that there are other tests available for testing association in GWAS [41], [42]. Linear regression has the advantage of simplicity in implementation across cohorts in large meta-analyses, and is able to incorporate covariates and interactions. Our method can be extended to other types of allelic effects: multiplicative, dominant and recessive, by computing the relevant expected values such as those in Table 1. Our work is restricted by other model assumptions which include independent random errors and normality of phenotype. For large sample sizes, linear regression can perform well with data which deviate far from normality [43].

Our results have important implications in practice. The methods of assessing the power of the sample size calculation in GWAS, which do not account for potential measurement errors, may optimistically over-estimate the power or equivalently under-estimate the sample size required. In the present study, we recommend the computation of sample size and power for GWAS of traits that have low repeatability, or differ between different grading scales and machinery, by a magnitude of more than 0.6 and 0.4 SD of true phenotype for comparison of means and variances respectively. A pilot study with multiple measurements is recommended to estimate the measurement error using our proposed method. This is to ensure accurate sample size calculation before GWAS. Finally, we note that the statistical power incorporating measurement errors is straightforward to compute using any software that provides values under the F distribution probability density function and the R code is available at request from the authors.

## Supporting Information

Text S1 **Derivation of squared correlation coefficient for comparison of phenotypic means and variability.**
(DOC)

Text S2 **Detailed quality control procedures for SCES samples genotyped on OmniExpress chips.**
(DOC)

Text S3 **Estimation of measurement error using repeated measurements.**
(DOC)
