# Supplementary material for: Impact of Measurement Error on Testing Genetic Association with Quantitative Traits
Source: PLoS One. 2014 Jan 24;9(1):e87044. doi: 10.1371/journal.pone.0087044 (PMC3901720; doi:10.1371/journal.pone.0087044)
Supplement: Text S1 — Derivation of squared correlation coefficient for comparison of phenotypic means and variability. (DOC) [file pone.0087044.s001.doc]

**Text S1**

**Comparison of phenotypic means**

We derive the expressions for used to compute the power to detect differences in means. Based on the definition of correlation,

$$\rho^{2}=\frac{[Cov(X,Y)]^{2}}{Var(X)Var(Y)}$$

For comparison of means, the covariance and variance are as follows:

$$\begin{matrix} Cov(X,Y)=E(XY)-E(X)E(Y) \\ =2p(1-p)\beta+4\beta p^{2}-2p[2p(1-p)\beta+2\beta p^{2}]=2p(1-p)\beta\\ Var(X)=2p(1-p) \end{matrix}$$

Hence, $\rho^{2}=\frac{(2p(1-p)\beta)^{2}}{2p(1-p)Var(Y)}=\frac{2p(1-p)\beta^{2}}{Var(Y)}$

$$E(Y)=E(X\beta)=2p\beta$$

**Comparison of phenotypic variability**

Based on the definition of correlation, for the comparison of variances of phenotypes with measurement error,

$$\rho^{2}=\frac{[Cov(Y_{e}^{2},X)]^{2}}{Var(X)Var(Y_{e}^{2})}$$

Using Table 1,

$$\begin{matrix} Cov(X,Y_{e}^{2})=E(XY_{e}^{2})-E(X)E(Y_{e}^{2}) \\ =2p(1-p)(1+\beta_{v}+\sigma^{2})+2p^{2}(1+2\beta_{v}+\sigma^{2})-[2p(1-p)+2p^{2}](1+2p\beta_{v}+\sigma^{2}) \\ =2p\beta_{v}(1-p) \\ Var(X)=2p(1-p) \\ Var(Y_{e}^{2})=E(Y_{e}^{4})-[E(Y_{e}^{2})]^{2} \\ =3(1-p)^{2}(1+\sigma^{2})^{2}+6p(1-p)(1+\beta_{v}+\sigma^{2})^{2}+3p^{2}(1+2\beta_{v}+\sigma^{2})^{2}-(1+2p\beta_{v}+\sigma^{2}) \\ =2[(1+\sigma^{2})^{2}+4p\beta_{v}(1+\sigma^{2})+p\beta_{v}^{2}(3+p)] \end{matrix}$$

Hence,

$$\rho^{2}=\frac{p(1-p)\beta_{v}^{2}}{(1+\sigma^{2})^{2}+4p\beta_{v}(1+\sigma^{2})+p\beta_{v}^{2}(3+p)}$$
