# Supplementary material for: Impact of Measurement Error on Testing Genetic Association with Quantitative Traits
Source: PLoS One. 2014 Jan 24;9(1):e87044. doi: 10.1371/journal.pone.0087044 (PMC3901720; doi:10.1371/journal.pone.0087044)
Supplement: Text S2 — Detailed quality control procedures for SCES samples genotyped on OmniExpress chips. (DOC) [file pone.0087044.s002.doc]

**Text S2**

**Detailed quality control procedures for SCES samples genotyped on OmniExpress chips**

We excluded 20 individuals based on: sample call-rates of less than 95% and excessive heterozygosity (n=10), cryptic relatedness (n=7), gender discrepancies (n=2), and population admixture (n=1). This left a total of 615 samples for statistical analysis, of which 609 samples had complete data for BP, age, gender and BMI. During SNP QC, 2,357 SNPs had high missingness (>5%), 723 SNPs had genotype frequencies deviated from HWE (*p* value <10^-6^), and 117 772 SNPs were monomorphic or had MAF<1%. These SNPs were removed, yielding 589,316 genotyped SNPs for association analysis.
