# Supplementary material for: Impact of Measurement Error on Testing Genetic Association with Quantitative Traits
Source: PLoS One. 2014 Jan 24;9(1):e87044. doi: 10.1371/journal.pone.0087044 (PMC3901720; doi:10.1371/journal.pone.0087044)
Supplement: Text S3 — Estimation of measurement error using repeated measurements. (DOC) [file pone.0087044.s003.doc]

**Text S3**

**Estimation of measurement error using repeated measurements**

For the comparison of means, our model can be re-formatted as two normal distributions

$Y_{i}|Y_{i}^{*}\sim N(Y_{i}^{*},\sigma_{u}^{2})$, $Y_{i}^{*}\sim N(\mu+\beta X_{i},\sigma_{\varepsilon}^{2})$,

where $Y_{i}$ is the observed measurement, $Y_{i}^{*}$is the unobserved measurement without measurement error for the *i*th individual, $\sigma_{u}^{2}$ is variance of the measurement error and $\sigma_{\varepsilon}^{2}$ is the natural variability of measurement. Then, our observed measurement is$Y_{i}\sim N(\mu+\beta X_{i},\sigma_{\varepsilon}^{2}+\sigma_{u}^{2})$. The two variances are nondifferential and not easily separable. However, it is possible to estimate each variance when multiple measurements are available. Assuming we have *k* repeated measurements for each individual, the conditional distribution for the *i*th average is $\overline{Y_{i}}|Y_{i}^{*}\sim N\left(Y_{i}^{*},\frac{\sigma_{u}^{2}}{k}\right)$ and after integrating the unobserved measurement without error we can obtain the distribution of observed measurement . Using any one reading (i.e. *m*th), the distribution is $Y_{i,m}\sim N(\mu+\beta X_{i},\sigma_{\varepsilon}^{2}+\sigma_{u}^{2})$. Hence, $\sigma_{\varepsilon}^{2}$ and $\sigma_{u}^{2}$ become differential and can be estimated separately by taking the difference of the variance of residuals from these two linear regressions. Using one reading, we can estimate the total variance and using the average of repeated measurements, the estimate of can be obtained from the variance of residuals. By performing two regressions with different numbers of measurement, we are able to estimate the measurement error. The same can be extended to comparison of variances model.
